# Therapeutic Effects of Saponins for the Prevention and Treatment of Cancer by Ameliorating Inflammation and Angiogenesis and Inducing Antioxidant and Apoptotic Effects in Human Cells

**DOI:** 10.3390/ijms231810665

**Published:** 2022-09-14

**Authors:** Muhammad Imran Khan, Gul Karima, Muhammad Zubair Khan, Jin Hyuk Shin, Jong Deog Kim

**Affiliations:** 1 Department of Biotechnology, Faculty of Biomedical and Life Sciences, Kohsar University, Murree 47150, Pakistan; drimrankhan@kum.edu.pk or imranbiotech1@gmail.com; 2 Department of Bionanotechnology, Graduate School, Hanyang University, Seoul 04763, Republic of Korea; karimaali99@gmail.com; 3 Department of Biotechnology, Chonnam National University, Yeosu 59626, Republic of Korea; zobiskhan143@gmail.com (M.Z.K.); geobae@biolsystems.com (J.H.S.); 4 Research Center on Anti-Obesity and Health Care, Chonnam National University, Yeosu 59626, Republic of Korea

**Keywords:** apoptosis, cytotoxicity, inflammation, NRF-2, saponins, IL-1β

## Abstract

Saponins are natural compounds found in plants and have a diverse range of applications. However, the therapeutic potential of saponins in regulating cytotoxicity, angiogenesis, and inflammation in mammalian cells is yet to be explored. Here, we investigated the therapeutic effects of saponins from green tea by exploring the cytotoxic effects of saponins by inducing apoptosis in the human cancer cell lines hepatocellular carcinoma (HEPG2) and colorectal adenocarcinoma (HT29). The anti-angiogenesis effect of saponins was also investigated in human umbilical vein endothelial cells (HUVEC). We explored the ability of saponins to attenuate inflammation in a dose-dependent manner in normal human cells. It was found that saponins exhibit cytotoxic effects in cancer cells and not in normal cells at the same concentration. Cytotoxicity was measured by inducing apoptosis by enhancing caspase-3 (cas-3) activation and B-cell lymphoma-2 (*Bcl-2*)-associated X protein (*BAX*) gene expression and suppressing the antiapoptotic protein, Bcl-2. The inhibition of HUVEC proliferation was due to the suppression of the phosphoinositide 3-kinase (PI3K), protein kinase B (*AKT*), vascular endothelial growth factor receptor-2 (*VEGFR-2*), and nuclear factor kappa B (*NF-κB*). We also observed the antioxidant potential of green tea-derived saponins against free radicals in reactive oxygen species (ROS)-induced cells. Here we observed that the saponins exhibited free radical scavenging activities and activated nuclear factorerythroid 2-related factor 2 (NRF-2) leading to the upregulation of antioxidant-related genes in human embryonic kidney 293 (HEK293) cells. Furthermore, we demonstrated that the anti-inflammatory effects were due to the suppression of pro-inflammatory cytokines interleukin (IL)-1β, IL-6, tumor necrosis factor-alpha (TNF-α), and inducible nitric oxide synthase (iNOS) in HEK293 cells. The significance of the work is we are the first to report on the anti-cancer effects of saponins based on the anti-inflammatory, antioxidant, anti-angiogenesis, and apoptosis induction properties. In conclusion, green tea-derived saponins could be effective therapeutics for the treatment of cancer.

## 1. Introduction

Cancer is a complex chronic illness that causes early death worldwide [1,2]. It is defined by faulty signaling mechanisms that result in uncontrolled cellular proliferation. A lack of programmed cell death, apoptosis, is a major factor in both cancer development and the response to treatments [3]. Numerous gene families regulate apoptotic pathways intrinsically or extrinsically [4,5,6]. Apoptosis is activated by extracellular or intracellular signals, which set off a series of events that lead to DNA fragmentation and nuclear condensation. During apoptosis, the apoptosome interacts with apoptotic protease activating factor 1 (Apaf-1) and procaspase-9 to produce an active form of caspase-9. The intrinsic apoptotic pathway depends on the release of cytochrome c from mitochondria to form the apoptosome [7]. Both pro- and anti-apoptotic proteins and genes regulate apoptosis and advance this biological process. 

Anti-apoptotic substances have a detrimental effect and prevent apoptosis [8]. Although many anti-cancer medicines primarily use chemotherapy to promote apoptosis, several anti-apoptotic medications have been linked to the development of treatment resistance in cancers and other side effects. Finding effective medications that target the apoptotic signal transduction pathway is necessary to improve cancer therapies’ clinical outcomes [9]. 

Infection, injury, or exposure to pollutants is stimuli that can cause inflammation, one of the main immune response processes [10]. The body uses inflammation as a defense mechanism against these stimuli. However, chronic inflammation can lead to the onset of several conditions, including rheumatoid arthritis, cardiovascular disease, diabetes, obesity, inflammatory bowel disease, asthma, and central nervous system (CNS)-related conditions like Parkinson’s disease and amyotrophic lateral sclerosis (ALS) [11]. The oxidative stress associated with chronic inflammation contributes to various disorders, including Parkinson’s, Alzheimer’s, diabetes, cancer, hypertension, septic shock, asthma, arthritis, and arteriosclerosis. As a result, there has been increased interest in the management of pro-inflammatory processes and oxidative stress induced during chronic inflammation for ameliorating or curing serious illnesses [12,13]. 

Antioxidants neutralize pro-oxidant molecules and thus modify the oxidant–antioxidant profile of bodily systems [14]. Antioxidants are linked to decreased reactive oxygen species (ROS) and free radical generation, as well as decreased susceptibility to lipid peroxidation, oxidative stress, post-translational protein modification, and DNA damage. The majority of these protective antioxidants can be found in food and plants [15]. Numerous drug discovery efforts continue to take advantage of plants as an abundant source of therapeutic molecules, and many natural products have been used to generate anti-cancer medicines [16].

Many natural compounds, especially alkaloids, are employed in clinical settings and are frequently used in ethnopharmacology. Plant-based natural remedies have been used for generations to treat a wide range of illnesses. In the United States, the use of chemicals produced from bioactive plants is expanding due to the accessibility, potency, and lack of documented side effects; natural products are typically the first line of defense in the management of acute and chronic illnesses [17,18,19]. In plants, various phytochemicals work together to produce pharmacological effects. Alkaloids, polyphenols, terpenoids, and flavonoids, like anthocyanin and flavone, have produced anti-inflammatory and antioxidant effects.

However, many of these bioactive chemicals exhibit potent cytotoxicity [20]. Drugs used to treat cancer are toxic to healthy, quickly dividing cells. Another significant issue is the development of cancer cell resistance to chemotherapy medications. Therefore, combining different medications may be more effective in combating these unwanted side effects [21]. The tumor cells’ apoptotic pathways must be activated by any effective cancer treatment plan. Numerous natural substances identified as prospective sources of novel anticancer medications have been shown to trigger apoptosis in cancer cells [22]. 

Numerous plants contain saponin, a steroid or triterpene glycoside. Numerous saponins are found in green tea leaves, including theasaponin B1, assamsaponin J, isotheasaponin B1–B3, and foliatheasaponin I–V. Neutrophil-stimulating [23], antibacterial, anti-inflammatory [24], antihypertensive [25], and anti-allergic activities have been reported in pharmacological research on tealeaf saponins [26]. We have previously stated that a green tea leaf extract (TE) rich in saponins and free of catechins prevented diet-induced hypercholesterolemia in rats [27]. Therefore, this study focused on TE-derived saponins and their ability to alleviate inflammation and angiogenesis, and induce antioxidant and apoptotic effects in cancer cells. The aim of the current study was to investigate the therapeutic effects of green tea isolated saponins for inhibiting and treating human cancers.

## 2. Results

### 2.1. Identification, Structure Elucidation, and Quantification of the Isolated Compounds

**Figure 1:**
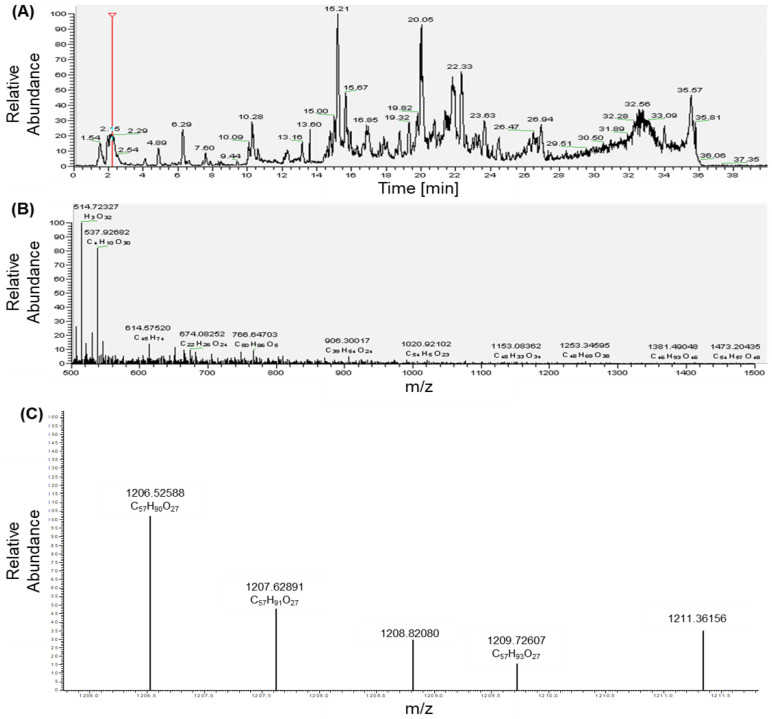
LC/TOF-MS analysis of the isolated saponins. (**A**) The LC/MS chromatogram of the saponin fraction isolated from green tea seed ethanolic extracts. (**B**) The MS of the saponin fraction isolated from green tea seed ethanolic extracts. (**C**) The NMR of the saponins detected and identified in the saponin fraction purified from green tea seed ethanolic extracts.

Saponin-rich fractions were isolated and purified from green tea seed ethanolic extracts by resin column chromatography followed by preparative HPLC. The purified saponin fractions were subjected to time-of-flight liquid chromatography and mass spectrometry (LC/TOF-MS) and nuclear magnetic resonance (NMR) for identification, structure elucidation, and quantification of the present compounds (Figure 1). The saponin fraction mainly consist of theasaponin E1 with small quantities of theasaponin E3 and theasaponin A and B (Figure S1).

### 2.2. Cytotoxic Effects of Saponins 

**Figure 2:**
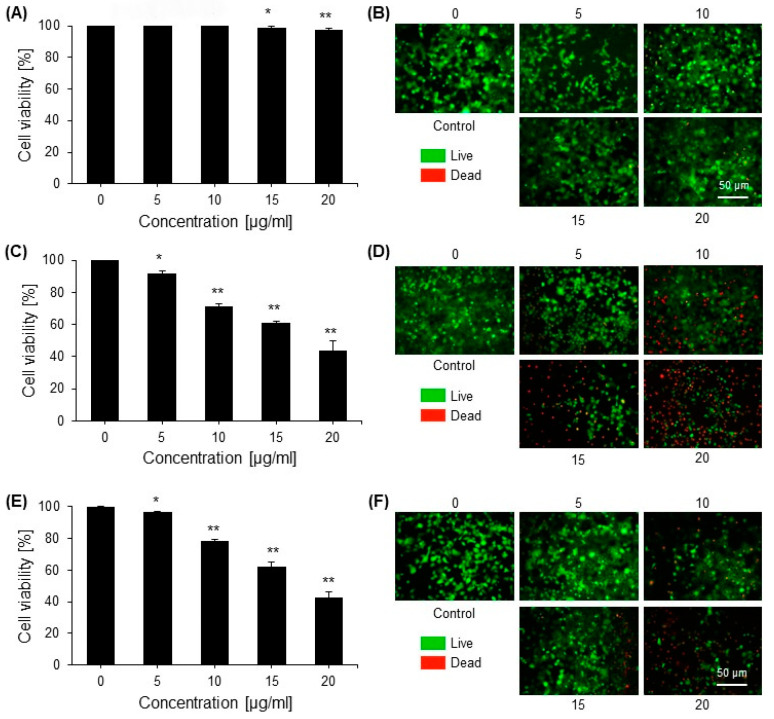
The cytotoxic effects of saponins on normal cells (HEK293) and cancer cell lines (HEPG2 and HT29) were determined via a cell viability assay. (**A**) The cytotoxic effect of saponins on normal cells (HEK293) was determined via an MTT assay. (**B**) HEK293 cell viability under various concentrations of saponins was assessed using calcein-AM (green) and propidium iodide (PI; red) double staining. Representative confocal images of live and dead cells are shown. (**C**) The cytotoxic effect of saponins on the hepatic carcinoma cell line (HEPG2) was determined via an MTT assay. (**D**) HEPG2 cell viability under various concentrations of saponins was assessed using the live and dead cell determination kit. Representative images of the control and treated cells are shown. (**E**) The cytotoxic effect of saponins on HT29 cells was determined by an MTT assay. (**F**) HT29 cell viability under various concentrations of saponins was assessed using the live and dead cell determination kit. Representative images of the control and treated cells are shown. The data are shown as the mean ± standard error of the mean (SEM) from three independent experiments (*n* = 3). * = *p* < 0.01 and ** = *p* < 0.001 compared with the control.

The toxicity of the saponins isolated from green tea seed extract was determined by an MTT assay. Safe and toxic doses were determined first in a viability test using normal cells (HEK293). It was found that saponins are safe at the maximum dose rate of 25 µg/mL with a cell viability of 90%. The cytotoxic effect of saponins at the non-toxic concentration was determined by an MTT assay in normal cells and in cancer cells. The MTT assay for cytotoxicity showed that saponins exhibited a strong dose-dependent cytotoxic effect in cancer cells (Figure 2).

### 2.3. Anti-Angiogenesis Effect of Saponins

**Figure 3:**
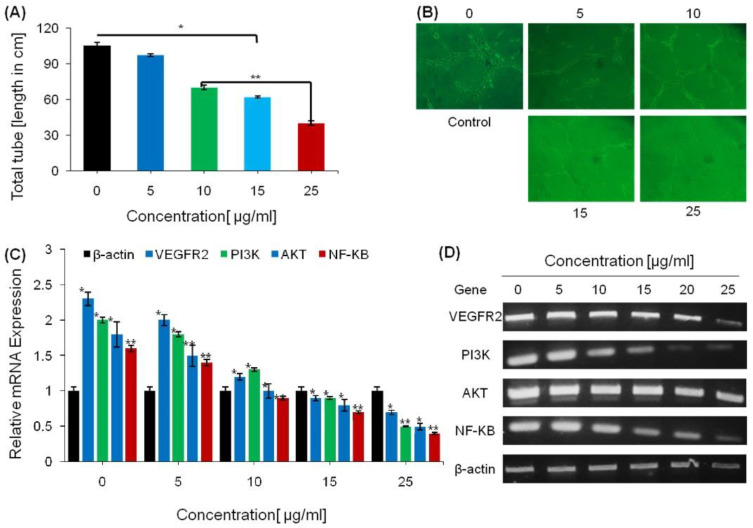
Effects of saponins on angiogenesis suppression. (**A**) HUVEC proliferation and formation of tubes on a Matrigel was calculated and presented as the total tube length in control and treated groups. (**B**) Photographs of HUVEC proliferation and tube formation in the control and treated groups. (**C**) The effect of saponins on the expression of angiogenesis-related genes determined by RT-PCR. (**D**) Gel electrophoresis of the PCR products from the treated and control cells. The data are shown as the mean ± SEM of three separate experiments. * = *p* < 0.01 and ** = *p* < 0.001 compared with the control.

The effect of green tea seed saponins was examined for endothelial cell tube formation by a quantitative analysis of the tube length formed on a Matrigel. The purified fraction of saponins exhibited a significant inhibitory effect on capillary tube formation in a dose-dependent manner when HUVECs were supplemented with increasing doses (5, 10, 15, and 25 µg/mL) of saponins. The lowest concentration of saponins significantly inhibited tubular structure formation, and complete disruption of capillary tubes was observed at 25 µg/mL. These results demonstrate that green tea saponins effectively inhibit HUVEC proliferation and tube formation on a Matrigel. The total tube length was significantly decreased with the increasing doses of saponin compared with the control cells’ tube length. The inhibitory effects of saponins on HUVEC proliferation and capillary tube formation on a Matrigel were noticeable on the images taken of the cells for each dose concentration (Figure 3). 

The effects of saponins onVEGFR-2expression and the PI3K/AKT/ERK pathway was further investigated in HUVECs using the safe concentration ranges of saponins. The regulatory effect on gene expression was determined by RT-PCR using gene-specific primers (Table 1).

Saponins reduced VEGFR-2 and vascular endothelial (VE)-cadherin complex expression via NF-κB suppression, a critical growth factor for proliferation and vascular remodeling. Various growth factors are well documented to play important roles in the PI3K/AKT/ERK pathway activation. The RT-PCR results showed that AKT and ERK mRNA levels were suppressed via VEGFR-2 down-regulation in a dose-dependent manner. Treatments with saponins led to significant reductions in PI3K, AKT, ERK, VEGFR-2, NF-κB, and β-Catenin mRNA expression (Figure 3). 

### 2.4. Free Radical-Scavenging Effects of Saponins

**Figure 4:**
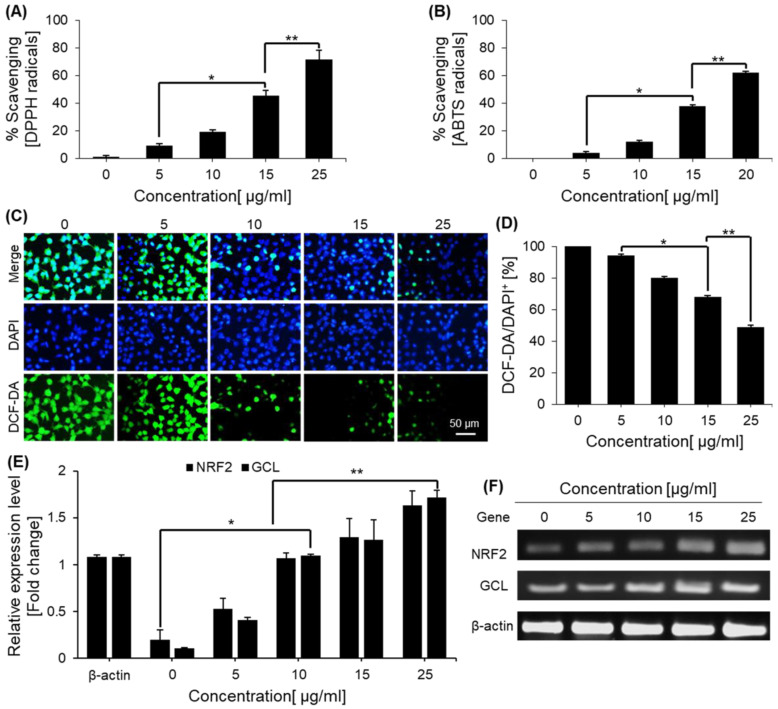
Antioxidant potential of saponins against free radicals, ROS, and the expression of antioxidant genes in HEK293 cells. (**A**) Free radical scavenging activities of saponins against DPPH free radicals. (**B**) Free radical scavenging activities of saponins against ABTS free radicals. (**C**) Determination of H2O2-induced cellular ROS in control and saponin-treated cells using dichlorodihydrofluorescein diacetate (DCFDA) staining under a fluorescence microscope. (**D**) DCFDA-positive cells per 4′,6-diamidino-2-phenylindole (DAPI)-positive cells were quantified in the control and saponin-treated cells. (**E**) Antioxidant-associated gene expression was determined in treated and control cells determined RT-PCR. (**F**) Gel electrophoresis of the PCR products from the antioxidant-related gene RT-PCR. The data are shown as the mean ± SEM of three separate experiments. * = *p* < 0.01 and ** = *p* < 0.001 compared with the control.

The free radicals α, α-diphenyl-β-picrylhydrazyl(DPPH), and 2,2′-azino-bis(3-ethylbenzothiazoline-6-sulfonic acid (ABTS) were used to determine the free radicals scavenging potential of green tea seed saponins. Figure 4 shows the saponins’ free radical scavenging activity against DPPH and ABTS. Saponins showed effective free radical savaging against DPPH and ABTS in a dose-dependent manner.

### 2.5. Saponins Effect on Intercellular ROS Levels

The antioxidant effect of saponins against intercellular ROS was determined by dichlorofluorescein diacetate (DCFDA) staining using HEK293 cells. The results revealed that the ROS, detected by DCFDA, markedly decreased with the saponin treatment in a dose-dependent manner (Figure 4). However, the DCFDA-positive to 4′,6-diamidino-2-phenylindole (DAPI)-positive cell ratio was greater in the untreated control cells. We analyzed the mRNA expression ofNRF2 and its target, glutamate cysteine ligase (GCL), by RT-PCR to investigate whether saponins affected H2O2-induced ROS generation in hepatocytes. These factors are involved in regulating ROS production. NRF2 and GCL mRNA levels increased dose-dependently with treatments of saponins (Figure 4). Therefore, these data showed that saponins reduced oxidative stress and inhibited ROS generation.

### 2.6. Pro-Apoptotic Effects of Saponins

**Figure 5:**
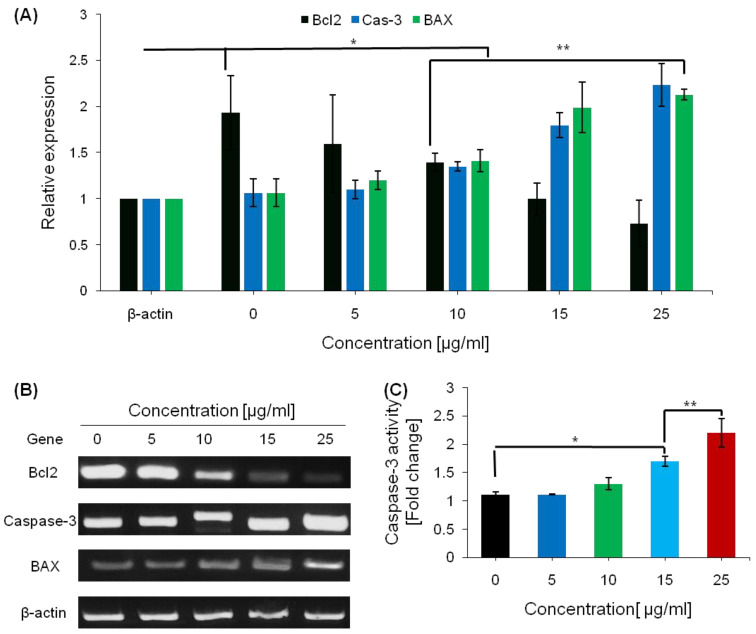
The effect of saponins on the induction of apoptosis in HEPG2 cells. (**A**) The effect of saponins on the expression of BAX and caspase-3 determined via RT-PCR. (**B**) Gel electrophoresis of the PCR products from the apoptosis promoting genes RT-PCR. (**C**). The effects of saponins on the activity of caspase-3 in HEPG2 cells were determined by a calorimetric analysis. The data are shown as the mean ± SEM from three independent experiments. * = *p* < 0.001, ** = *p* < 0.001 compared with the control.

In order to determine the pro-apoptotic effects of saponins in cancer cells, HEPG2 cells were treated with various concentrations of saponins, and the expression of several apoptosis-related genes was measured by RT-PCR. After treating the cells with various concentrations of saponins, caspase-3 activity was found to be dose-dependently increased (Figure 5C).

Saponins elevated the mRNA expression of apoptosis-promoting genes B-cell lymphoma-2 (Bcl-2)-associated X protein (BAX) and caspase-3 (Figure 5). The greatest increase in mRNA expression of apoptosis-promoting genes occurred 48 h after treatment with the saponins.

Since treatment with the saponins increased the caspase-3 expression and apoptosis in this study, we investigated whether Bcl-2 family members were involved in the increase in apoptosis. In HEPG2 cells, apoptosis was induced by licochalcone C (LC) and the expression of Bcl-2 was analyzed after the treatment with the saponins. Compared with the control group, treatments with saponins resulted in a concentration-dependent reduction in the mRNA level of Bcl-2 (Figure 5). 

### 2.7. Effects of Saponins on Proinflammatory Cytokines 

**Figure 6:**
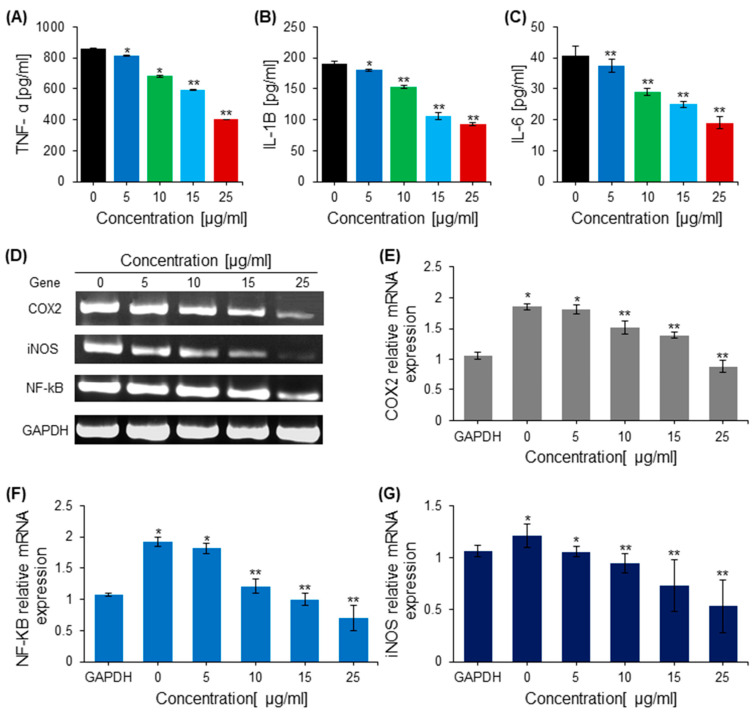
The anti-inflammatory effect of saponins. (**A**) Quantification of Tumor necrosis factor alpha (TNF-α) in cells treated with various concentrations of saponins. (**B**) The effect of saponins on the release of the pro-inflammatory cytokine interleukin-1β (IL-1β), quantified in control and treated cells with an ELISA. (**C**) The effect of saponins on the release of the proinflammatory cytokine IL-6, quantified in control and treated cells with an ELISA. (**D**) The DNA bands of inflammation-related genes from the RT-PCR. (**E**). The relative mRNA expression of the pro-inflammatory gene COX-2 in treated and control cells determined by RT-PCR. (**F**) The relative mRNA expression of the pro-inflammatory gene NF-kB in treated and control cells determined by RT-PCR. (**G**) The relative mRNA expression of the pro-inflammatory gene iNOS in treated and control cells determined by RT-PCR. * = *p* < 0.001, ** = *p* < 0.001 compared with the control.

The effect of saponins on the suppression of inflammatory cytokines interleukin (IL)-1β, IL-6, tumor necrosis factor-alpha (TNF-α), and iNOS in HEK293 cells was analyzed using ELISA kits specific for inflammatory cytokines. Saponins were found to decrease the levels of inflammatory cytokines IL-1B dose-dependently and TNF-α compared with the untreated control cells (Figure 6). HEK293 cells were subjected to various concentrations of saponins for 24 h to determine the effects of the saponins on the mRNA expression of proinflammatory cytokines using RT-qPCR. Treatment with saponins reduced the expression of inflammatory cytokine mRNA in a dose-dependent manner compared with the untreated control cells (Figure 6).

## 3. Discussion

Natural products from plants, like alkaloids, are highly cytotoxic to many cancer cells via various mechanisms. Many of these compounds have been developed into anti-cancer drugs, such as vinblastine, vincristine, camptothecin, taxol, and ellipticine [28]. These compounds exhibit cytotoxicity against cancer cell lines A549 (human lung carcinoma), BGC-823 (human gastric carcinoma), BEL-7402 (human liver carcinoma), HTC-8 (human colon carcinoma), and A2780 (human ovarian carcinoma) [29]. Moreover, the anti-cancer properties of natural plant products are related to their anti-proliferative, pro-apoptotic [30], and topoisomerase II inhibitory effects. Natural plant products have been shown to result in the overexpression of the apoptosis-related proteins p53 and BAX and the suppression of Bcl-2 [31,32]. Here we reported that treatment with saponins exhibited strong cytotoxic effects in hepatic carcinoma cells (HEPG2) and human colon cancer cells (HT29). However, saponins showed a less toxic effect in normal human kidney cells (HEK293) using the same concentrations. The cytotoxic effects of saponins may be due to the pro-apoptotic effects in the cancer cell lines. The development of pharmaceuticals that selectively initiate the apoptotic machinery within tumor cells could be an effective anti-cancer treatment [33]. In this study, apoptosis was initiated HEPG2 cells treated with saponins. The extent of apoptosis induction appears to be saponin dose-dependent. The activation of BAX and the inhibition of Bcl-2 results in mitochondrial disruption and the subsequent release of cytochrome c through the outer mitochondrial membrane into the cytosol. Inside the cytosol, cytochrome c associates with Apaf-1 and activates caspase-9, which triggers the activation of caspase-3 [34,35]. Activated caspase-3 functions as a key inducer of apoptosis and leads to the cleavage and inactivation of vital cellular substrates.

Since tumor metastasis occurs through angiogenesis, angiogenesis inhibition is one of the most prominent therapeutic strategies for cancer treatment. Therefore, we inhibited the angiogenesis-promoting signaling via treatments with saponins. Saponins reduced VEGFR-2 and VE-cadherin complex expression via NF-κB downregulation. VEGFR-2 and VE-cadherin are important growth factors for proliferation and vascular remodeling. Various growth factors have been found to play an important role in the activation of the PI3K/AKT/ERK pathway [31]. RT-PCR results showed that AKT and ERK mRNA expression was reduced via VEGFR-2 downregulation in a dose-dependent manner. Treatment with the highest nontoxic concentration of saponins (25 µg/mL) led to significant decreases in PI3K, AKT, ERK, VEGFR-2, NF-κB, and β-catenin mRNA expression. These results provide a molecular basis for understanding the green tea seed-derived saponins inhibition of angiogenesis.

Free radicals have deleterious and hazardous effects on various biomolecules, including DNA, lipids, and proteins causing membrane peroxidation, inflammation, mutation, and cancer. Scavenging these free radicals is crucial to neutralize the deleterious effects and maintain homeostasis. Antioxidants from natural products need to be explored and investigated further. We investigated the free radical scavenging potential of green tea-derived saponins. Saponins showed radical scavenging against various free radicals. Since saponins can scavenge free radicals, thereby preventing lipid oxidation via a chain-breaking reaction, they could serve as potential pharmaceuticals and nutraceuticals. 

In addition, we investigated whether saponins can affect H2O2-induced ROS generation in hepatocytes. We focused on the transcription factor NRF2 and GCL. NRF2 is a potential regulator of cell resistance to oxidants, and it exhibits multiple cell-protective effects against a range of toxicities and chronic diseases associated with oxidative stress [36]. When cells are exposed to oxidative stress, GCL is rapidly activated by NRF2, contributing to NRF2-mediated cell protection following oxidative stress [37]. Therefore, we show that saponins activated and increased the expression of these antioxidant enzymes, which alleviated the oxidative stress in cells.

Inflammation is a major cause of several impaired cellar processes and the imbalance of homeostasis, leading to severe complications, and is linked to various illnesses and diseases. Therefore, it is crucial to attenuate inflammation to sustain the normal physiology of the cells.

Saponins attenuated inflammation by inhibiting the NF-κB signaling pathway. Our previous study reported that saponin alleviated neuroinflammation in glial cells [38]. This study’s results suggest that saponins modulate hepatic injury and fibrosis by attenuating inflammation. It has been reported that natural products like melittin inhibit liver failure via blocking NF-κB signaling and apoptotic pathways in the D-galactosamine/LPS-induced mouse liver failure model [39,40]. Therefore, saponins are an effective natural product for treating and preventing various illnesses and diseases with their anti-inflammatory and antioxidant effects and attenuating cancer by exerting antiangiogenic and pro-apoptotic effects. The significance of the work is that we are the first to report on the anti-cancer effects of saponins based on their anti-inflammatory, antioxidant, anti-angiogenesis, and apoptosis induction properties.

## 4. Materials and Methods

### 4.1. Cell Lines and Conditions

The human umbilical vein endothelial cell (HUVEC; CRL-1730™),HEPG2 (HEPG2; HB-8065), HT29 (HTB-38™), and HEK293 (CRL-1573™) line was used for the treatment purposes. HUVECs were grown in Endothelial Cell Growth Basal Medium-2 [EBM-2] media (Clonetics, Walkersville, MD, USA) supplemented with EGM-2 Single Quot Kit (Clonetics, USA). HEPG2 and HT29 cells were cultured in Dulbecco’s modified Eagle medium (DMEM; Sigma-Aldrich, St. Louis, MO, USA) with 10% Fetal Bovine Serum (FBS; Life Technologies, Carlsbad, CA, USA), and 1% Penicillin/Streptomycin (P/S; Life Technologies). HEK293s were cultured in McCoy’s 5a Medium Modified (Sigma; 200-655-4) supplemented with 10% FBS and 1% P/S. All cells were cultured at 37 °C in humidified conditions with 95% O_2_ and 5% CO_2_.

### 4.2. MTT Assay

An MTT assay was used to measure the cytotoxicity of saponins. Briefly, 5 × 10^4^ cells were seeded in a 96-well plate with 100 µL of Roswell Park Memorial Institute (RPMI) medium supplemented with 5% fetal bovine serum (FBS). Various concentrations of saponins were introduced after 24 h. Fifty microliters of MTT (5 mg/mL stock solution) were added after 48 h, and the plates underwent an additional 4 h of incubation. The formazan blue that developed in the cells was removed from the medium and dissolved in 100 µL dimethyl sulfoxide (DMSO). The optical density was determined at 540 nm.

### 4.3. Cell Viability Assay

The LIVE/DEAD cell imaging kit (Thermofisher, Waltham, MA, USA) was used to measure the vitality of the cells following the manufacturer’s instructions. Briefly, 5 × 10^3^ immortalized hepatocytes were grown in a 96-well plate and treated with H2O2 for 48 h. After three washes with phosphate buffered saline (PBS), the cells were treated for 30 min in an incubator with a solution of calcein-AM and propidium iodide in PBS. Samples were imaged using a confocal microscope (LSM 800; Zeiss Osfieldern Germany) after they were rinsed twice with PBS. The number of live and dead cells was recorded in five separate confocal microscope images from each experiment.

### 4.4. HUVECs Tubes Formation Assay

The anti-angiogenic properties of saponins were measured in an in vitro angiogenesis experiment using HUVECs. Matrigel (BD Bioscience, Bedford, MA, USA)-coated 96-well microtiter plates were used to cultivate the cells at 37 °C. Matrigel-coated wells were filled with HUVEC suspensions in medium (1 × 10^4^ cells per well) and then incubated for 4 h at 37 °C with 5% CO_2_. The wells were treated with various concentrations of saponins, which were then further incubated for 4 h. Tube formation was observed and captured using a phase contrast inverted microscope (Nikon, Tokyo, Japan). Using Image J version 1.53k (NIH, Bethesda, MD, USA) software with an angiogenesis analyzer, the tube length was examined from five images taken from randomly selected cell culture fields in each well. The length of the entire tube was calculated and evaluated compared with the controls. The experiments were done twice with three replicates for each experiment.

### 4.5. Angiogenesis-Associated mRNA Expression

The effect of saponins on the mRNA expression of angiogenesis-promoting signaling proteins was assessed using RT-PCR. HUVECs were seeded onto a six-well plate at a density of 9.6 × 10^4^ cells per well. The cells were grown in complete endothelial cell growth basal medium (EBM-2) up to 70% confluency. The cells were then treated with various concentrations of saponins and incubated for 24 h. Following the manufacturer’s instructions, the total RNA was isolated from the cells using an RNA extraction kit (Qiagen, LLC—Germantown, MD, USA). A total of 1 g of RNA was used to synthesize cDNA using the RevertAid Premium First Strand cDNA Synthesis Kit (Thermo Scientific, Waltham, MA, USA).

### 4.6. DPPH Radical Scavenging Activities

The DPPH free radical scavenging activity of saponins was determined using a method previously described by Hu et al. with slight modifications [41]. About 0.2 mL of aqueous saponin solutions at various concentrations were added to 2 mL of the DPPH solution (21.4 µg/mL in ethanol). The reaction was protected from light and incubated for 30 min at room temperature. After incubation, the absorbance was measured at 517 nm. The sample solution was replaced with a 70% ethanol solution to obtain the baseline absorbance level of the DDPH alone (A0). DPPH was added to the samples in a similar manner to obtain the ADPPH. The percentage of free radical scavenging activity was calculated using the following equation:

% Radical scavenging activity = 1 − [(ADPPH − ASample)/A0] × 100%(1)

where ASample represents the absorption of the sample without DPPH.

### 4.7. ABTS Radical Scavenging Activities

The antioxidant activity was evaluated using a method previously described by Hwang et al. [42]. Briefly, solutions of saponins were prepared in different concentrations (100–1000 μg/mL). The saponin solutions (50 μL) were mixed with the ABTS+ solution (4.95 mL) and incubated for 1 h, protected from light. The mixture was analyzed at 734 nm using a Spectro UV–VIS Double Beam plate reader (Model UVD-3500, Labomed, Inc., Los Angeles, CA, USA). TROLOX was used to create the standard curve. The results were in TROLOX equivalent (TE)/gm. The percentage of inhibition was calculated using the following equation:

Inhibition (%) = 100% × (Acontrol − Asample)/Acontrol(2)

where Acontrol represents the control reaction absorbance, and Asample represents the saponin solution absorbance.

### 4.8. Reactive Oxygen Species (ROS) Assay 

The ROS production was measured using the ROS-sensitive dye, 2′,7′-dichlorodihydrofluorescein diacetate (H2DCFDA). H2DCFDA reacts with peroxyl products and peroxyl radicals but not with singlet oxygen directly. However, singlet oxygen rapidly forms peroxyl radicals and thus can indirectly contribute to dichlorodihydrofluorescein (DCF) formation. For the measurement of endogenous ROS levels in HEK293 cells, cells were incubated with 10 μM DCFDA (Molecular Probes, Inc., Eugene, OR) for 30 min and stained with DAPI. The samples were observed under an inverted fluorescence microscope (IX53; Olympus, Japan). DCF fluorescence intensity was examined in the images, taken during a single session with the exposure and gain settings kept constant, of samples obtained from one experiment that was processed side-by-side. The number of DCF- positive cells among all cells stained with DAPI was counted in three random fields of view (*n* = 3). 

### 4.9. mRNA Expression Level of the NRF2 and GCL Genes

The total RNA was extracted from immortalized hepatocyte cells using a TRIzol kit (Life Technologies, Carlsbad, CA, USA) following the manufacturer’s protocol. The concentration of the RNA samples was measured using a Nanodrop ND-1000 (Thermo Fisher Scientific). For RT-PCR analysis, cDNA was transcribed from 100 ng of DNase I-treated RNA using SuperScript III Reverse Transcriptase (Thermo Fisher Scientific) and Oligo DT (Roche Diagnostics, Rotkreuz, Switzerland). RT-PCR amplification reactions were carried out in a detection system from Bio-Rad (Hercules, CA, USA) in a 20 μL volume. Gene expression was analyzed by performing RT-PCR amplification with SYBR green (Dyne Bio) and the specific primers for the housekeeping gene glyceraldehyde-3-phosphate dehydrogenase (GAPDH) and antioxidant marker genes NRF-2 and GCL.

### 4.10. Caspase-3 Activity Assay

The caspase-3 activity was assessed by the cleavage of DEVD-p-nitroanaline (pNA) into pNA by caspase-3 using the Caspase-3 Colorimetric Assay kit (R&D Systems, Inc. Minneapolis, MN, USA). The HEPG2 cells were incubated with various concentrations of LC (0, 25, 35, and 45 µg/mL). The cells were washed with PBS and suspended in a lysis buffer (20 mM HEPES, pH 7.9, 20% glycerol, 200 mM KCl, 0.5 mM EDTA, 0.5% NP40, 0.5 mM DTT, and 1% protease inhibitor cocktail [Sigma-Aldrich, St. Louis, MO, USA]). Cell lysates (100 µg total protein) were added to 96-well plates with the DEVD-pNA at 37 °C for 1 to 2 h to determine the caspase-3 activity. The optical density of each well was measured at 405 nm using a fluorescence plate reader (Bio-Rad Laboratories, Inc). The experiment was done in three replicants for each experimental condition. 

### 4.11. RT-PCR for Apoptosis-Related and Pro-Inflammatory Genes

The total RNA was extracted from the HEPG2 cells treated with saponins after 48 h. The RNA samples were reverse transcribed for 30 min at 42 °C with the High-Capacity cDNA Reverse Transcription Kit (Applied Biosystems, Waltham, MA, USA). RT-PCR was performed using 1 μL of the cDNA, 2X SYBR-Green PCR Master Mix (Applied Biosystems), and 200 nm of forward and reverse apoptosis-related and pro-inflammatory gene primers (Table 1). Each assay was run on an RT-PCR system in triplicate, and the expression foldchanges were derived using the comparative CT method.

### 4.12. ELISA Quantification of Proinflammatory Cytokines

The concentrations of TNF-α, IL-1β, and IL-6 in HEK293 cells were measured using ELISA kits. The cells were cultured in a six-well plate and treated with various concentrations of saponins. The concentrations of the inflammatory cytokines were measured using cell lysate from treated and control cells following the manufacturer’s instructions.

### 4.13. Statistical Analysis

The data were expressed as the mean ± standard deviation (SD) and were analyzed using SPSS (Version 11.5; SPSS Inc., Chicago, IL, USA) and M.S. Office, Excel software. A one-way analysis of variance (ANOVA) was applied to observe the significance between the groups. Duncan’s multiple range tests (DMRTs) were performed to calculate the significant difference among the groups. A *p*-value of less than 0.05 was considered significant. 

## Figures and Tables

**Table 1:** Primer sequences used in RT-PCR.

No.	Gene	Primer Sequence	Accession Number	Size [bp]
1	COX2	CTTCCGATTGAAGCCCCCAT GGTCGTGTAGCGGTGAAAGT	KC753760	137
2	iNOS	GAGCAGAGATCGTGCCACTC TTGGTGGAATGGCAGGTAGG	AF045477	182
3	NF-kB	GGGCAGGAAGAGGAGGTTTC GCAGTGCCATCTGTGGTTGA	NM_001382627	600
4	NRF2	ATCTTCGAGGAGCTCACCCT TCAGTGTCTTGGGACTTGCC	AH010686	480
5	Caspase-3	TGTCCTGGGACACCGGTTAT TCTGTTGCCACCTTTCGGTT	AJ413269	646
6	GCL	CAGTGGTTTGCTATGCTGCG CCGGGGAATTCGATTCACTAC	AF198534	843
7	VEGFR2	CGGTCAACAAAGTCGGGAGA CAGTGCACCACAAAGACACG	EU826563	123
8	PI3K	TGGAGAGAGAGCAGTTCCAAT ATCTCTCGGCAGTCTTGTCG	NM_001256045	555
9	AKT	GAAGACGGGAGCAGGCG AAGGTGCGTTCGATGACAGT	NM_001382431	694
10	BCL-2	TCTCATGCCAAGGGGGAAAC CAATCCTCCCCCAGTTCACC	KY098799	629
11	BAX	CCAGAGGCGGGGGATGATTG GCAGGGTAGATGAATCGGGG	NM_001291430	461
12	β-actin	GGCTCTTTTCCAGCCTTCCT AATGCCAGGGTACATGGTGG	HQ154074	151
13	GAPDH	GCTCCCTCTTTCTTTGCAGC GTTGTCATGGATGACCTTGGC	JN613429	77

## Data Availability

Not applicable.

## Supplementary Materials

The following supporting information can be downloaded at: https://www.mdpi.com/1422-0067/23/18/10665/s1.
