# Correction: Khan et al. Therapeutic Effects of Saponins for the Prevention and Treatment of Cancer by Ameliorating Inflammation and Angiogenesis and Inducing Antioxidant and Apoptotic Effects in Human Cells. *Int. J. Mol. Sci.* 2022, *23*, 10665

**DOI:** 10.3390/ijms242015235

**Published:** 2023-10-16

**Authors:** Muhammad Imran Khan, Gul Karima, Muhammad Zubair Khan, Jin Hyuk Shin, Jong Deog Kim

**Affiliations:** 1 Department of Biotechnology, Faculty of Biomedical and Life Sciences, Kohsar University, Murree 47150, Pakistan or imranbiotech1@gmail.com; 2 Department of Bionanotechnology, Graduate School, Hanyang University, Seoul 04763, Republic of Korea; karimaali99@gmail.com; 3 Department of Biotechnology, Chonnam National University, Yeosu 59626, Republic of Korea; zobiskhan143@gmail.com (M.Z.K.); geobae@biolsystems.com (J.H.S.); 4 Research Center on Anti-Obesity and Health Care, Chonnam National University, Yeosu 59626, Republic of Korea

In the original publication by Khan et al., 2022 [1], there were mistakes in Figure 2 and Figure 6D as published. In Figure 2B,D,F, there was duplication of some images in the treatment and control groups for various cells. The duplicated images of cells in various groups were corrected and replaced with the original images. Also, in Figure 2A,C,E, the line bars showing the statistical analysis were wrongly placed on the graph bars. In Figure 6D, the gel band of COX2 was cropped from a wrong gel, and now it was replaced with the correct picture cropped from the original gel band of COX2. The corrected Figure 2 and Figure 6 appear below. 

The authors state that the scientific conclusions are unaffected. This correction was approved by the Academic Editor. The original publication has also been updated.

## Figures and Tables

**Figure 2:**
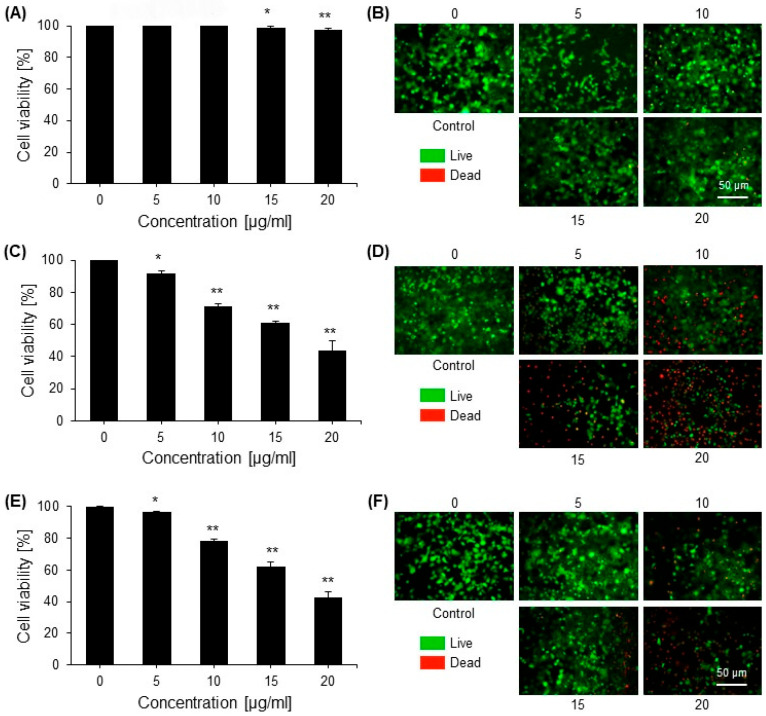
The cytotoxic effects of saponins on normal cells (HEK293) and cancer cell lines (HEPG2 and HT29) were determined via a cell viability assay. (**A**) The cytotoxic effect of saponins on normal cells (HEK293) was determined via an MTT assay. (**B**) HEK293 cell viability under various concentrations of saponins was assessed using calcein-AM (green) and propidium iodide (PI; red) double staining. Representative confocal images of live and dead cells are shown. (**C**) The cytotoxic effect of saponins on the hepatic carcinoma cell line (HEPG2) was determined via an MTT assay. (**D**) HEPG2 cell viability under various concentrations of saponins was assessed using the live and dead cell determination kit. Representative images of the control and treated cells are shown. (**E**) The cytotoxic effect of saponins on HT29 cells was determined by an MTT assay. (**F**) HT29 cell viability under various concentrations of saponins was assessed using the live and dead cell determination kit. Representative images of the control and treated cells are shown. The data are shown as the mean ± standard error of the mean (SEM) from three independent experiments (*n* = 3). * = *p* < 0.01 and ** = *p* < 0.001 compared with the control.

**Figure 6:**
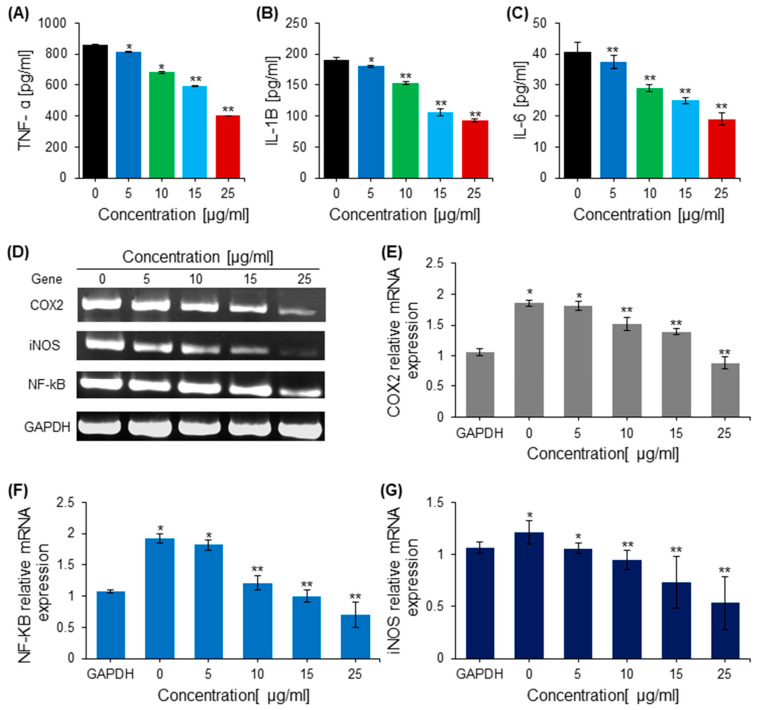
The anti-inflammatory effect of saponins. (**A**) Quantification of Tumor necrosis factor alpha (TNF-α) in cells treated with various concentrations of saponins. (**B**) The effect of saponins on the release of the pro-inflammatory cytokine interleukin-1β (IL-1β), quantified in control and treated cells with an ELISA. (**C**) The effect of saponins on the release of the proinflammatory cytokine IL-6, quantified in control and treated cells with an ELISA. (**D**) The DNA bands of inflammation-related genes from the RT-PCR. (**E**). The relative mRNA expression of the pro-inflammatory gene COX-2 in treated and control cells determined by RT-PCR. (**F**) The relative mRNA expression of the pro-inflammatory gene NF-kB in treated and control cells determined by RT-PCR. (**G**) The relative mRNA expression of the pro-inflammatory gene iNOS in treated and control cells determined by RT-PCR. * = *p* < 0.001, ** = *p* < 0.001 compared with the control.
