# Supplementary material for: Therapeutic Effects of Saponins for the Prevention and Treatment of Cancer by Ameliorating Inflammation and Angiogenesis and Inducing Antioxidant and Apoptotic Effects in Human Cells
Source: Int J Mol Sci. 2022 Sep 14;23(18):10665. doi: 10.3390/ijms231810665 (PMC9504392; doi:10.3390/ijms231810665)
Supplement: Supplementary file 1 [file ijms-23-10665-s001.zip › Copounds isolation and extraction process.pdf]

Saponins extraction process

Green tea (Camellia sinensis) seeds were collected from the Myungin Shin Gwang Su tea garden (Suncheon, Korea). The seeds were dried, de-hulled, and ground into powder. The powder (3 kg) was defatted with n-hexane (4 L) under sonication at 30°C for 5 h and then dried. The defatted seed powder was further extracted by refluxing with 70% ethanol at 60°C for 8 h. The resulting extract was filtered, concentrated using a rotatory vacuum evaporator (SB-100, Eyela), freeze dried, and weighed. The extract was again subjected to extraction with butanol and water mixture and concentrated with rotary rotatory evaporator. Saponins extraction from the crude extract was carried out by Non-polar macropours resins (D101). Resins were thoroughly washed 2 times with ethanol and then distal water. 10 g of the extract was dissolved in 30 ml double distal water and mixed with the washed resin and kept overnight at room temperature. 50 g of the extract was dissolved in 100 ml double distilled water and was passed through the resin column eluted first with 0.4 N NaOH followed by neutralization of the extract and resin mixture with HCL and again elution with eluted with 100% ethanol resulted in saponin rich mixture. This saponin mixture was then subjected to column chromatography using C18 column. First eluted with 10% MeOH to wash carbohydrates followed elution with 60% MeOH to wash out various acids and Finally with 100% MeOH to obtain the saponins mixture. Pure saponin (Theasaponin E1) was then isolated from this fraction by HPLC preparative high-performance liquid chromatography (HPLC) (Shimadzu Co., Kyoto, Japan) equipped with a photodiode array (PDA) detector. The extract was separated on a Luna C-18(2) reverse phase column (250 mm x 21.2 mm, 15 µm; Phenomenex, Inc., Torrance, CA, USA) at 35 °C. Solvent A was methanol and solvent B was distilled water containing 0.1% formic acid. The non-linear gradient system used was initially A/B (74:26) to A/B (74.8:25.2) at 33.5 min to A/B (100:0) for 2 min and held A/B (100:0) for 10 min and then A/B (74:26) for 12 min. Components were detected at 210 nm. Flow rate 7 mL/min was used. Identification and determination of the isolated saponins were done by LC/TOF-MSand NMR
